# The Regulation of Bacterial Biofilm Formation by cAMP-CRP: A Mini-Review

**DOI:** 10.3389/fmicb.2020.00802

**Published:** 2020-05-14

**Authors:** Cong Liu, Di Sun, Jingrong Zhu, Jiawen Liu, Weijie Liu

**Affiliations:** School of Life Sciences, Jiangsu Normal University, Xuzhou, China

**Keywords:** biofilm, second messengers, cAMP-CRP, c-di-GMP, carbon catabolite repression

## Abstract

Biofilms are communities of microorganisms that live in a self-produced extracellular matrix in order to survive in hostile environments. Second messengers, such as c-di-GMP and cAMP, participate in the regulation of biofilm formation. c-di-GMP is a major molecule that is involved in modulating the bacterial transition between a planktonic lifestyle and biofilm formation. Aside from regulating carbon catabolism repression in most bacteria, cAMP has also been found to mediate biofilm formation in many bacteria. Although the underlying mechanisms of biofilm formation mediated by cAMP-CRP have been well-investigated in several bacteria, the regulatory pathways of cAMP-CRP are still poorly understood compared to those of c-di-GMP. Moreover, some bacteria appear to form biofilm in response to changes in carbon source type or concentration. However, the relationship between the carbon metabolisms and biofilm formation remains unclear. This mini-review provides an overview of the cAMP-CRP-regulated pathways involved in biofilm formation in some bacteria. This information will benefit future investigations of the underlying mechanisms that connect between biofilm formation with nutrient metabolism, as well as the cross-regulation between multiple second messengers.

## Introduction

Biofilms are structured communities of microorganisms in which cells are embedded in an extracellular matrix (Flemming and Wuertz, 2019). Biofilms are generally formed to promote bacterial survival in harsh environments (Flemming et al., 2016). While biofilm formation is a complex process regulated by several different factors in various bacteria, some regulators of biofilm formation, such as second messengers, are common to nearly all bacteria (Kalia et al., 2013; Jenal et al., 2017). The second messenger c-di-GMP is universally recognized as a “switch molecule” that controls the bacterial transition between a planktonic lifestyle and biofilm formation (Dahlstrom and O’Toole, 2017). High c-di-GMP levels induce biofilm formation, whereas low c-di-GMP levels induce planktonic growth (Valentini and Filloux, 2016; Liu et al., 2017). Another well-characterized second messenger cAMP, the cofactor of CRP, is primarily involved in carbon catabolite repression in bacteria. In *Escherichia coli* and *Salmonella enterica* serovar Typhimurium, the uptake and concomitant phosphorylation of preferential carbon sources, such as glucose, requires the phosphoenolpyruvate: sugar phosphotransferase system (PTS) (Deutscher et al., 2006). When PTS sugars are in the medium, the phosphoryl group of phosphorylated EIIA protein (EIIA∼P) is transferred to PTS sugars in order to complete the uptake of these sugars into the cell (Hogema et al., 1997; Park et al., 2006). When PTS sugars are limited and the non-PTS sugars are present in the medium, adenylyl cyclase CyaA is activated directly by EIIA∼P to synthetize cAMP (Hogema et al., 1997; Deutscher et al., 2006; Park et al., 2006). Subsequently, cAMP-CRP activates the transcription of genes encoding these proteins for the transport of non-PTS sugars, which results in the transport of these non-PTS sugars into the cell (Park et al., 2006). Thus, carbon catabolite repression is achieved by cAMP-CRP (Deutscher et al., 2006). In addition, cAMP-CRP is an archetypical global regulator. For instance, this complex regulates the transcription of 7% of the genes in *E. coli*, which illustrates that the biological role of cAMP goes far beyond carbon catabolite repression and includes other cellular processes such as biofilm formation (Hufnagel et al., 2016).

It is widely accepted that second messengers play a central role in biofilm formation, and there are several reviews regarding the c-di-GMP-mediated regulation of biofilm formation (Ha and O’Toole, 2015; Purcell et al., 2016; Valentini and Filloux, 2016; Jenal et al., 2017). The underlying mechanisms of biofilm formation mediated by cAMP-CRP, however, also deserve to be reviewed. First, high cAMP levels influence biofilm formation differently among bacterial species. For example, cAMP-CRP promotes biofilm formation in *E. coli* and *Pseudomonas aeruginosa* (Müller et al., 2009; Sutrina et al., 2015), but inhibits biofilm formation in *Serratia marcescens* and *Vibrio cholerae* (Kalivoda et al., 2013). Moreover, cAMP-CRP is involved in diverse physiological functions, thus connecting physiological processes with biofilm formation (Sutrina et al., 2015, 2019). In this mini-review, we provide an overview of the ancillary role of cAMP-CRP-mediated biofilm formation in different bacteria. This information will benefit future investigations regarding the underlying mechanisms that connect between biofilm formation and other important physiological processes in bacteria.

## cAMP-CRP Regulates Biofilm Formation in *E. coli*

**FIGURE 1:**
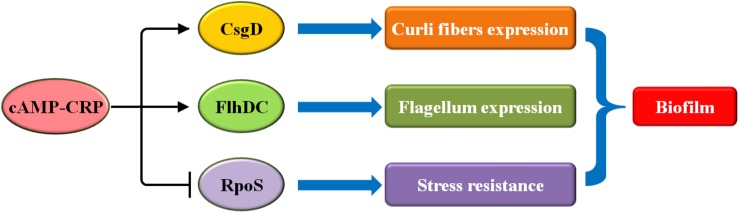
In *E. coli*, cAMP-CRP regulates biofilm formation though three pathways: CsgD, FlhDC, and RpoS. Arrowheads indicate activation, with the rear of the arrow representing inhibition.

Biofilm formation in *E. coli* is mediated by several carbon sources, including PTS and non-PTS sugars (Sutrina et al., 2015, 2019). Both sugars exert inhibitory effects on biofilm growth at high concentrations, which are reversed by the addition of exogenous cAMP (Sutrina et al., 2015, 2019). It has also been shown that cAMP-CRP, in response to carbon sources, plays an ancillary role in the regulation of biofilm formation in *E. coli* (Figure 1).

First, cAMP-CRP mediates curli fiber synthesis. Curli fibers, as the part of the extracellular matrix, are involved in the adhesion and attachment of bacteria to surfaces in order to initiate biofilm formation (Barnhart and Chapman, 2006). The synthesis of curli fibers is controlled, at least in part, by six proteins that are encoded by the *csgBA* and *csgDEFG* operons (Rudd, 2000; Barnhart and Chapman, 2006). CsgD is a major regulator that mediates the expression of both operons (Brombacher et al., 2003; Ogasawara et al., 2011). cAMP-CRP directly activates the transcription of *csgD*, thereby controlling biofilm formation (Hufnagel et al., 2016).

Second, cAMP-CRP regulates flagellum biosynthesis. The flagellum is a critical organelle involved in bacterial motility and chemotaxis (Xie et al., 2011; Guttenplan and Kearns, 2013). During biofilm formation in *E. coli*, the flagella act as a mechanosensory, sensing the solid surface signals and driving the initial attachment (Belas, 2014). Flagellum biosynthesis in *E. coli* involves approximately 50 gene clusters that are controlled by the master motility complex FlhDC (Chevance and Hughes, 2008; Guttenplan and Kearns, 2013). cAMP-CRP positively regulates the transcription of *flhDC*, thus mediating flagellum-associated physiological processes, including biofilm formation (Soutourina et al., 1999).

Third, cAMP-CRP directly inhibits *rpoS* transcription (Lange and Hengge-Aronis, 1994; Kalia et al., 2013). RpoS, the stationary-phase-specific sigma factor, governs many stress responses to protect *E. coli* from some specific harmful environments including starvation, osmotic shock, and so on (Loewen et al., 1998; Battesti et al., 2011). During biofilm formation in *E. coli*, RpoS critically inhibits the initiation of biofilm formation, especially during the exponential growth phase (Corona-Izquierdo and Membrillo-Hernández, 2002). Thus, cAMP-CRP promotes biofilm formation by repressing *rpoS* transcription. In conclusion, cAMP-CRP plays a major ancillary role in the regulation of biofilm formation in *E. coli.*

## cAMP-CRP Regulates Biofilm Formation in *P. aeruginosa*

**FIGURE 2:**
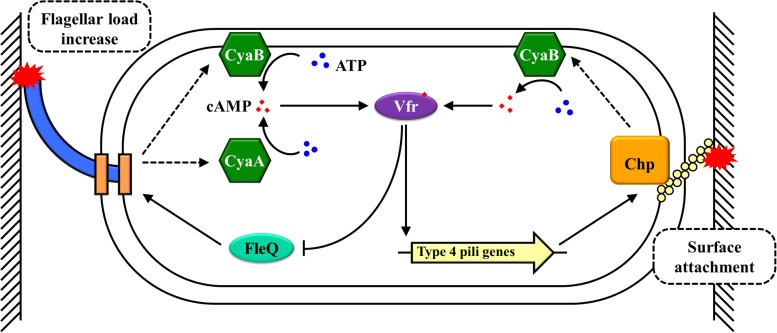
A solid surface signal regulates biofilm formation by cAMP-CRP in *P. aeruginosa*. Arrowheads indicate activation, with the rear of the arrow representing inhibition.

The involvement of cAMP in the biofilm formation of *P. aeruginosa* is well-established. In contrast to *E. coli*, the preferred carbon sources of *P. aeruginosa* are acetate or tricarboxylic acid (TCA) cycle intermediates rather than the hexoses, such as glucose and fructose, that are preferred by most γ-proteobacteria (Aranda-Olmedo et al., 2005; Deutscher et al., 2006). In *P. aeruginosa*, carbon catabolite repression is governed by preferred carbon sources; carbon catabolite repression may be regulated by different mechanisms, such as catabolite repression control (Crc) proteins instead of cAMP-CRP (Hester et al., 2000; Aranda-Olmedo et al., 2005). A virulence factor regulator (Vfr) shares a high degree of homology to *E. coli* CRP (67% sequence identity) and is found to be mainly involved in regulating the expression of a set of genes encoding extracellular virulence factors in *P. aeruginosa* (West et al., 1994; Gosset et al., 2004; Coggan and Wolfgang, 2011). Although cAMP is still the allosteric activator of Vfr, the cAMP-Vfr complex has functional differences from the cAMP-CRP complex in terms of response signals and regulatory mechanisms. The signals responded by cAMP-Vfr are calcium, high osmolarity, and solid surfaces rather than PTS or non-PTS sugars (Wolfgang et al., 2003; Rietsch and Mekalanos, 2006; Persat et al., 2015). In *P. aeruginosa*, the underlying mechanisms of cAMP-Vfr-mediated responses to solid surfaces and control biofilm formation are well-characterized (Figure 2). When swimming bacteria come in contact with a solid surface via their flagellum, it leads to an increase in the flagellar load, which allows these cells to become tethered to the solid surface (Schniederberend et al., 2019). This increase in flagellar load causes an interaction occurring between FlhF (a SRP-like GTPase that induces a single flagellum to assemble at the pole) and FimV (a polar organizer) (Schniederberend et al., 2019). This interaction activates CyaA and CyaB, increasing the intracellular cAMP in the tethered bacteria (Schniederberend et al., 2019). Subsequently, cAMP activates the transcription factor Vfr and cAMP-Vfr inhibits the expression of *fleQ*, which encodes the master regulator of flagellar biogenesis, thereby negatively mediating the expression of flagellar genes and decreasing flagellar synthesis (Dasgupta et al., 2002; Wolfgang et al., 2003). At the same time, cAMP-Vfr triggers the expression of type IV pili (TFP) genes (West et al., 1994; Beatson et al., 2002; Wolfgang et al., 2003). TFP is the other mechanosensor to further sense the solid surface signal, which activates the Chp chemosensory system by the direct interaction between PilA of the TFP and PliJ of the Chp chemosensory system, which directly stimulates CyaB activity to synthesize additional cAMP (Fulcher et al., 2010; Persat et al., 2015). Finally, cAMP-Vfr further promotes the expression of TFP genes and other virulence factor genes that are involved in the biofilm formation (Wolfgang et al., 2003; Diaz et al., 2011). Therefore, the solid surface is sensed by the rotating bacterial flagellum, and this irreversible attachment requires the inhibition of flagellar synthesis coupled with increased expression of the TFP (Figure 2). In this process, cAMP-Vfr is a core regulator that mediates and integrates these pathways in *P. aeruginosa.* While numerous environmental signals can induce biofilm formation, the direct and primary signal is a solid surface (Belas, 2014). In *P. aeruginosa*, the flagellum acts as a mechanosensor, sensing the solid surface signal directly; this stimulates the synthesis of cAMP (Schniederberend et al., 2019). cAMP-Vfr further stimulates biofilm-inducing pathways, thereby promoting biofilm formation (Persat et al., 2015).

While CRP is the only effector of cAMP in most bacteria, a novel cAMP receptor, CbpA, has been identified in *P. aeruginosa* (Endoh and Engel, 2009). Although the Vfr-dependent functions are not influenced by the deletion of *cbpA*, CbpA, which is located at the cellular poles, may be critical to the polar flagellum in a cAMP-Vfr-dependent manner (Endoh and Engel, 2009). Additionally, it has been demonstrated that glucose starvation induces *P. aeruginosa* biofilm dispersal in a cAMP-Vfr-dependent manner (Huynh et al., 2012).

In most investigations, cAMP-Vfr promotes biofilm formation in *P. aeruginosa* (Luo et al., 2015; Persat et al., 2015). However, several studies have reported conflicting results regarding cAMP-Vfr regulating biofilm formation in *P. aeruginosa*. Some studies have shown that a mutant with a deleted *cpdA* gene, which encodes the CpdA protein that degrades cAMP in bacteria, had a defect in biofilm formation as compared to wild type (Ono et al., 2014; Almblad et al., 2019). Furthermore, cAMP-Vfr-inhibited *P. aeruginosa* biofilm formation depended on several phosphodiesterases (PDEs), which are c-di-GMP degrading enzymes; the primary PDEs in the cell include DipA, RbdA, and BifA (Almblad et al., 2019). At the same time, it was shown that elevated levels of c-di-GMP in *P. aeruginosa* caused an increase in biofilm formation by reducing cAMP levels (Almblad et al., 2015). Therefore, cAMP-CRP-mediated effects on biofilm formation were produced via multiple regulation pathways in *P. aeruginosa.*

## cAMP-CRP Regulates Biofilm Formation in Other Bacteria

In other members of the *Enterobacteriaceae* family, cAMP-CRP-mediated biofilm formation differs from that in *E. coli* and is often complicated. For example, in *S. marcescens*, cAMP-CRP suppresses, instead of promoting, biofilm formation by inhibiting type 1 fimbriae synthesis (Kalivoda et al., 2008, 2013). Furthermore, although the underlying mechanisms of biofilm formation in *S. enterica* serovar Typhimurium are similar to those reported in *E. coli*, but in contrast to *E. coli*, cAMP-CRP downregulates the transcription of *csgD* in *S. enterica* serovar Typhimurium, which promotes pellicle formation and inhibits biofilm formation (Paytubi et al., 2017). In *Klebsiella pneumoniae*, cAMP-CRP promotes biofilm formation by increasing type 3 fimbriae synthesis, which is indirectly via the c-di-GMP signaling pathways (Lin et al., 2016; Ou et al., 2017; Panjaitan et al., 2019). In summary, cAMP-CRP stimulates biofilm formation in *E. coli* and *K. pneumoniae* but inhibits biofilm formation in *S. marcescens*. Despite this discrepancy, some of the regulatory effects of cAMP-CRP on biofilm formation are common across the *Enterobacteriaceae*. First, in most *Enterobacteriaceae*, cAMP-CRP controls biofilm formation by regulating the synthesis of flagella or fimbriae, which is associated with motility (Soutourina et al., 1999; Kalivoda et al., 2008; Panjaitan et al., 2019). Second, the regulatory effects of cAMP-CRP on biofilm formation are dependent on carbon sources, due to carbon catabolite repression (Jackson et al., 2002; Sutrina et al., 2015, 2019).

In addition to the *Enterobacteriaceae* and *P. aeruginosa*, cAMP-CRP regulates biofilm formation in many other bacteria (He et al., 2007; Fong and Yildiz, 2008; Harrison et al., 2019; Ritzert et al., 2019). In *Yersinia pestis*, cAMP-CRP is also a central mediator of carbon catabolite repression. Although cAMP-CRP does not directly stimulate biofilm formation in this species, cAMP-CRP may indirectly promote *Y. pestis* biofilm production by facilitating the alternate carbon source expression profile, in part by regulating the carbon storage regulator protein CsrA, and stimulating another global regulator PhoP (Willias et al., 2015; Liu et al., 2016; Ritzert and Lathem, 2018; Ritzert et al., 2019). *V. cholerae* has a carbon catabolite repression system similar to that of *Enterobacteriaceae*, which is also regulated by cAMP-CRP (Houot and Watnick, 2008). Moreover, cAMP-CRP inhibits biofilm formation in *V. cholerae*. First, cAMP-CRP activates HapR, a master regulator that suppresses biofilm formation (Fong and Yildiz, 2008; Luo et al., 2015; Sutrina et al., 2019). Second, cAMP-CRP controls biofilm formation via the c-di-GMP signaling pathways (Fong and Yildiz, 2008; Krasteva et al., 2010; Kalia et al., 2013). Biofilm maturation in *V. cholerae* requires the production of extracellular matrix components, including matrix proteins and *Vibrio* polysaccharide (VPS) (Casper-Lindley and Yildiz, 2004). Two *vps* operons are expressed as a direct result of stimulation by two positive transcriptional regulators, VpsR and VpsT, both of which are c-di-GMP effectors; the activation of these operons increases biofilm formation in *V. cholerae* (Casper-Lindley and Yildiz, 2004; Zamorano-Sánchez et al., 2015). c-di-GMP does not alter the DNA-binding ability of VpsR. However, without c-di-GMP, the VpsR and RNA polymerase enzyme complex will not interact correctly with DNA and will not successfully generate an active transcription complex (Srivastava et al., 2011; Hsieh et al., 2018). VpsT is a typical c-di-GMP effector, and c-di-GMP is required for the activation of VpsT and the subsequent regulation of both *vps* operons (Krasteva et al., 2010). VpsR and VpsT can also activate each other, while cAMP-CRP negatively regulates both *vpsR* and *vpsT* (Fong and Yildiz, 2008). Thus, cAMP-CRP controls biofilm formation via the c-di-GMP signaling pathways in *V. cholerae*.

With the exception of *P. aeruginosa*, in which cAMP-Vfr may be exclusively involve in pathogenicity, virulence, and biofilm formation, cAMP-CRP-mediated biofilm formation in most bacteria primarily depends on the cAMP-CRP-dependent pathways related to carbon metabolism. Thus, the ancillary regulation of cAMP-CRP on biofilm formation by cAMP-CRP is associated with or dependent on the carbon metabolism in many bacteria.

## Conclusion and Perspectives

Biofilms have emergent properties that protect their inhabitants’ survival in hostile environments, such as those in which the available nutrients have changed (Myszka and Czaczyk, 2009; Zhang et al., 2014; Liu et al., 2017, 2019; Wang et al., 2017; Flemming and Wuertz, 2019). Several studies have shown that multiple bacteria form biofilms in response to changes in the types or concentrations of the available carbon sources (Myszka and Czaczyk, 2009; Zhang et al., 2014; Liu et al., 2017). The relationship between nutrient metabolism and biofilm formation is largely unknown. It is also unclear whether carbon molecules act as simple signals, stimulating biofilm formation, or whether the pathways associated with carbon metabolism are cross-regulated with those regulating biofilm formation. As a critical factor in carbon catabolite repression, cAMP-CRP may be the “connecter” between the carbon metabolism and biofilm formation. This possibility should be addressed in future investigations.

Second messengers play an important role in various physiological processes in bacteria. The involvement of the second messenger c-di-GMP in biofilm formation is well established in multiple bacteria (Matsuyama et al., 2016; Liu et al., 2017), and regulatory effects of c-di-GMP on biofilm formation have been carefully reviewed (Christen et al., 2010; Dahlstrom and O’Toole, 2017; Krasteva and Sondermann, 2017). Compared to c-di-GMP, which participates in the whole biofilm cycle (from initial attachment to dispersion), the regulatory effects of cAMP-CRP on biofilm formation seem mostly ancillary. However, the underlying mechanisms should also be considered. Indeed, a few studies have investigated the cross-regulation between c-di-GMP and cAMP-CRP. In *K. pneumoniae*, *P. aeruginosa*, and *V. cholerae*, cAMP-CRP/Vfr regulates the gene expression levels of PDE and DGC (c-di-GMP diguanylate cyclase) or the pathways associated with c-di-GMP (Fong and Yildiz, 2008; Lin et al., 2016; Almblad et al., 2019). However, the underlying mechanisms remain largely unknown. Future investigations should aim to characterize the pathways associated with the cross-regulation between cAMP and c-di-GMP.

## Author Contributions

CL, DS, JZ, and JL conducted the literature study and wrote the draft manuscript. WL edited and revised the manuscript.

## Conflict of Interest

The authors declare that the research was conducted in the absence of any commercial or financial relationships that could be construed as a potential conflict of interest.
